# Automatic detection and counting of wheat seedling based on unmanned aerial vehicle images

**DOI:** 10.3389/fpls.2025.1665672

**Published:** 2025-10-21

**Authors:** Hecang Zang, Yanjing Wang, Yilong Peng, Shaoyu Han, Qing Zhao, Jie Zhang, Guoqiang Li

**Affiliations:** ^1^ Institute of Agricultural Information Technology, Henan Academy of Agricultural Sciences, Zhengzhou, China; ^2^ Huanghuaihai Key Laboratory of Intelligent Agricultural Technology, Ministry of Agriculture and Rural Areas, Zhengzhou, China; ^3^ College of Life Sciences, Zhengzhou Normal University, Zhengzhou, China

**Keywords:** UAV, wheat seedling count, object detection, deep learning, density enhancement encoder

## Abstract

Wheat is an important food crop, wheat seedling count is very important to estimate the emergence rate and yield prediction. Timely and accurate detection of wheat seedling count is of great significance for field management and variety breeding. In actual production, the method of artificial field investigation and statistics of wheat seedlings is time-consuming and laborious. Aiming at the problems of small targets, dense distribution and easy occlusion of wheat seedling in the field, a wheat seedling number detection model (DM_IOC_fpn) combining local and global features was proposed in this study. Firstly, the wheat seedling image is preprocessed, and the wheat seedling dataset is built by using the point annotation method. Secondly, the density enhanced encoder module is introduced to improve the network structure and extract local and global contextual feature information of wheat seedling. Finally, the total loss function is constructed by introducing counting loss, classification loss, and regression loss to optimize the model, so as to enable accurate judgment of wheat seedling position and category information. Experiment on self-built dataset have shown that the root mean square error (RMSE) and mean absolute error (MAE) of DM_IOC_fpn were 2.91 and 2.23, respectively, which were 1.78 and 1.04 lower than the original IOCFormer. Compared with the current mainstream object detection models, DM_IOC_fpn has better counting performance. DM_IOC_fpn can accurately detect the number of small target wheat seedling, and better solve the problem of occlusion and overlapping of wheat seedling, so as to achieve the accurate detection of wheat seedling, which provides important theoretical and technical support for automatic counting of wheat seedlings and yield prediction in complex field environment.

## Introduction

Wheat is the world’s largest grain crop in terms of sowing area, yield and distribution. In 2022, according to FAO data, the global wheat planting area is 21.9 million hectares, with a yield of 80.8 million tons (FAO, 2023). The number of wheat seedling is an important evaluation indicator that reflects the quality of wheat sowing, growth status and yield trait (Liu et al., 2017). However, the current acquisition of wheat seedling mainly relies on manual field measurement, which have disadvantages such as poor timeliness, low efficiency and time-consuming and laborious processes, and can’t longer meet the needs of high-throughput screening in wheat breeding. With the continuous advancement of computer vision and artificial intelligence technology, especially the powerful ability of deep learning in automatic detection and processing of wheat seedling images, various counting problems of crop can be solved to achieve accurate and efficient crop counting.

At present, ground hyperspectral data acquisition is not only time-consuming and labor-consuming, but also difficult to effectively complete large-scale crop seedling monitoring due to the large number of bands and complex data processing; Although the remote sensing satellite data with high spatial resolution can cover a large area, the acquisition cost is high, which makes it difficult to ensure the accuracy of crop seedling information extraction, and there is great uncertainty. In contrast, the small and light Unmanned Aerial Vehicle (UAV) has become an important research hotspot and frontier direction in the field of crop seedling number information extraction by virtue of its advantages such as flexible and fast, non-destructive monitoring, high spatial resolution and imaging free from atmospheric interference, and its ingenious integration of the accuracy of ground hyperspectral data and the wide area coverage ability of satellite remote sensing. With the development of UAV remote sensing technology, crop image data with centimeter accuracy can be quickly obtained, and crop growth can be monitored in real time through image analysis and processing (Bai et al., 2023). Combined with crop growth model and historical data, crop yield can be scientifically predicted, which provides timely and accurate decision-making basis for agricultural production management. The combination of UAV remote sensing technology and artificial intelligence technology has brought revolutionary changes to the agricultural field (Sharma et al., 2021). With the continuous progress of technology, the future application prospects in crop production, disease management, breeding optimization and other aspects will be broader, bringing more accurate and efficient solutions for agricultural production (Lu and Young, 2020). In terms of data acquisition and processing, image quality control, occlusion processing and complex background separation have become difficult problems to improve the accuracy of recognition and counting (Li et al., 2024). From the perspective of cost-effectiveness, although the initial investment is relatively high, the long-term operation cost is far lower than that of traditional methods, and the use of UAV reduces the physical interference to farmland, which is conducive to sustainable agricultural practice.

Deep learning technology is widely used in agriculture, especially for crop target counting using image data. The crop target counting method mainly include detection based method (Joseph et al., 2015; Zhao et al., 2021; Lu et al., 2022), regression based method (Girshick et al., 2013) and density map based method (Jiang et al., 2021). Detection based method estimate the number of target by detecting object instances. However, this method only perform well in sparse and relatively large scale scenes, and perform poorly in dense and crowded scenes. The regression based method considers the detection target as a whole and learns the mapping relationship between low-level feature and the number of target to complete the counting task, but it requires manual feature extraction and isn’t suitable for small target detection. The density map based method takes learning the relationship between image features and their corresponding density maps as the starting point, generates predicted density map through gaussian kernel function, and then integrates the density map. The integrated result is the number of predicted target. At present, the representative detection methods mainly include broad learning (BL) (Ma et al., 2019), multicolumn convolutional neural network (MCNN) (Susheela Devi and Meena, 2017), convolutional social relation network (CSRNet) (Li et al., 2018), spatial/channel-wise attention regression networks (SCAR) (Gao et al., 2019), point-to-point network (P2PNet) (Song et al., 2021), and distribution matching for crowd counting (DM-Count) (Wang et al., 2020).

In recent years, many scholars have applied computer vision technology to crop detection, mainly focusing on improving the accuracy of detection algorithm, expanding the range of detection type, and optimizing algorithm to adapt to crop counting under different environmental condition. Zang et al. (2022b) extracted wheat coverage based on UAV images at seedling stage and constructed the relationship between coverage and plant density, coefficient of determination (R^2^) was 0.82. Wilke et al. (2021) proposed a new method to obtain multispectral images by UAV and evaluate the density of grain plant, with R^2^ of 0.83. Zang et al. (2022a) proposed YOLOv5s spike detection method based on improved attention mechanism, with accuracy rate of 71.61%, which better solved the problem of occlusion and overlapping of spike. Wen et al. (2024) proposed a generalized model for accurate counting of wheat spike in complex scene, average accuracy was 81.54%. Zhao et al. (2022) proposed a deep learning method for spike detection, with average accuracy of 90.5%. Zhao et al. (2023) proposed WheatNet to detect wheat spike from filling to maturity, the average accuracy of spike detection in filling stage was 90.1%, and the average accuracy of spike detection in maturity stage was 88.6%. Zaji et al. (2023) proposed a new enhancement algorithm for wheat spike counting, the mean absolute error (MAE) and root mean square error (RMSE) were 2.085 and 2.695, respectively. Although wheat spikes and wheat seedlings originate from organs at different growth stages of wheat, they both face core challenges such as small targets and dense distribution in dense target detection tasks. Therefore, the methods and ideas proposed in wheat spike detection research have important reference significance for wheat seedling detection. Wheat spikes are located at the top of the plant and are relatively independent targets, suitable for scenes in the middle and late stages of growth; whereas wheat seedlings are continuously and densely distributed in the field, easily obscured by leaves, and are mainly suitable for early growth stage detection. This study focuses on the difficult problem of wheat seedling detection and optimizes the model design specifically to adapt to its unique characteristics. In the images taken by UAV, wheat seedling is in the stage of two leaves at one heart, and the directions are different, so the horizontal detection can’t provide accurate direction information. However, there are many problem in the wheat seedling images taken by UAV. The wheat seedling is small, densely distributed, seriously occluded and overlapped with each other, which makes it difficult for the deep convolution network to extract the characteristics of small target wheat seedling, resulting in false detection and missed detection in wheat seedling detection. The current common target detection models can’t solve the above problems, and the detection effect of small targets and dense targets is poor. Therefore, it is necessary to further study and improve the deep convolution network.

This study proposes a wheat seedling detection model DM_IOC_fpn that integrates local and global feature, which can accurately detect small target wheat seedling in complex field environment, and better solve the problem of wheat seedling occlusion and overlap. The main purposes of this study is: (1) the wheat seedling image dataset is built; (2) the density enhanced encoder module is introduced to improve the network structure; (3) a wheat seedling number detection model DM_IOC_fpn was proposed; (4) The DM_IOC_fpn detection method is compared with the mainstream detection method.

## Materials and methods

### Study area

**Figure 1:**
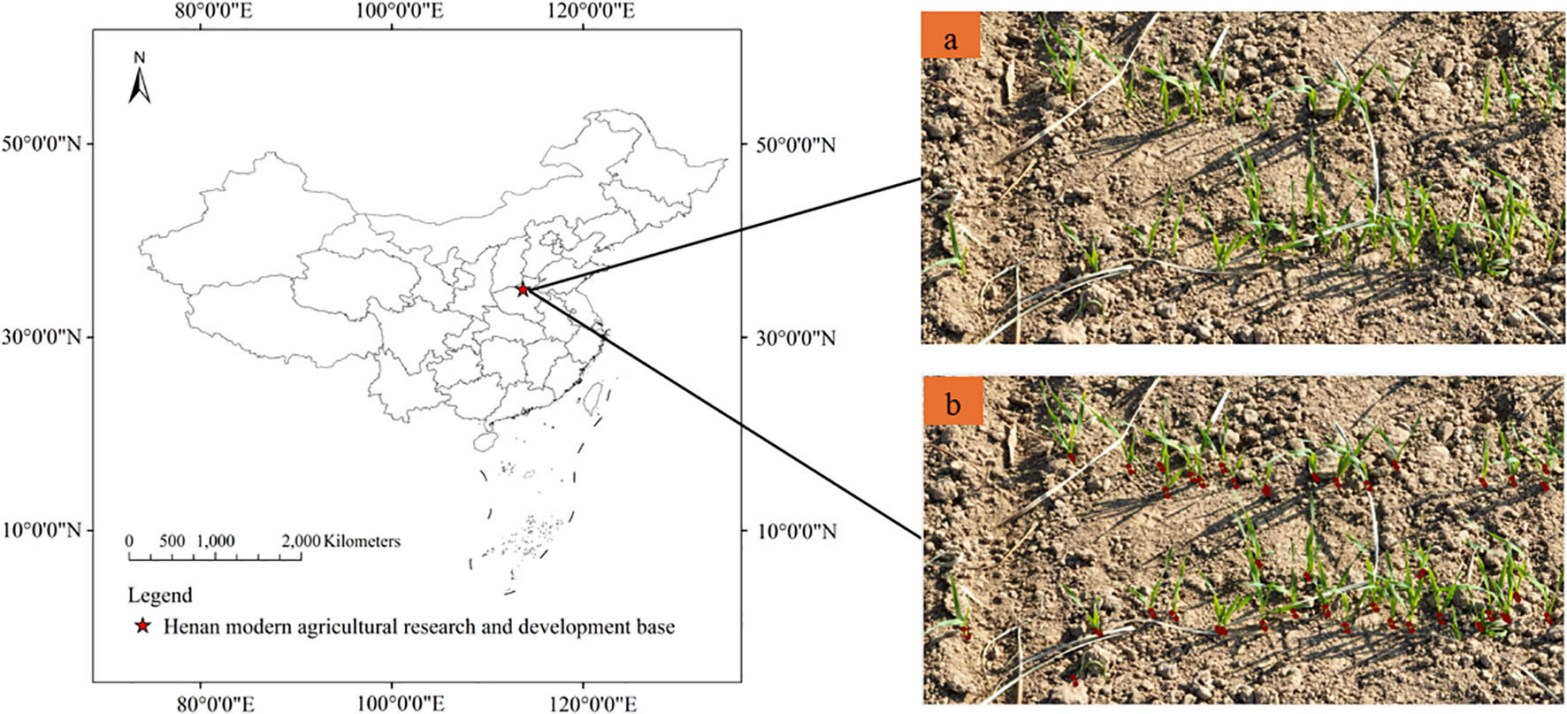
Study area and UAV images of wheat seedling **(a)** original image **(b)** annotation image.

The wheat regional test was conducted in Henan Modern Agricultural Research and Development Base of the Henan Academy of Agricultural Sciences, as shown in **Figure 1**. The climate type of the region is semi-arid and semi humid warm temperate continental monsoon climate, with an annual average temperature of 14.5°C and an average annual rainfall of 660 mm, mainly concentrated in June-September. The experiment was conducted in a completely randomized block design with three repetitions, six rows of new wheat varieties were planted in each plot. The sowing date was October 18, 2023. The test materials were 80 wheat varieties, and the plot area was 12 m^2^.

### UAV images of wheat seedling

**Figure 2:**
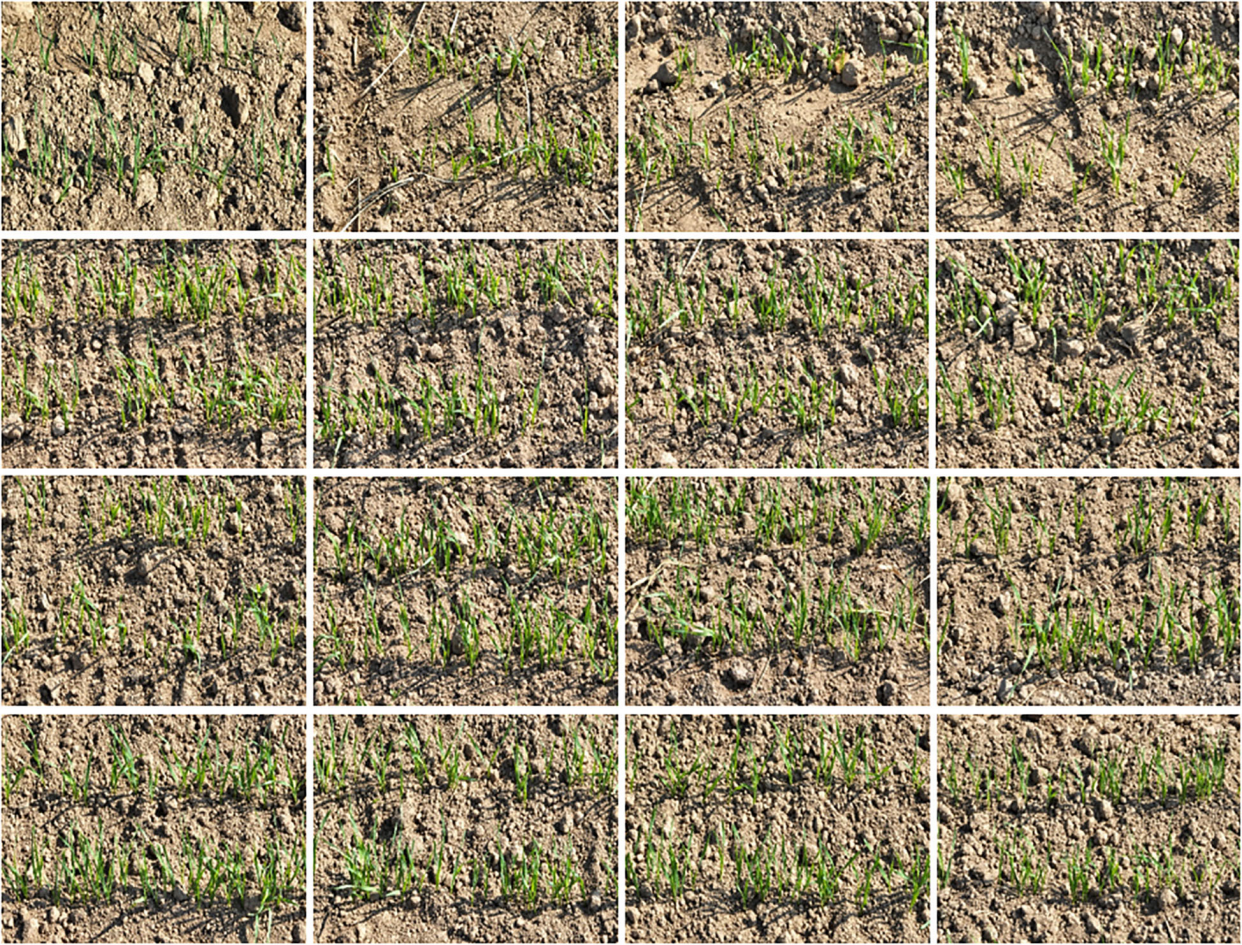
Example of partial images of wheat seedling stage.

DJI Mavic 3 UAV was used in the experiment. The camera pixel was 20million pixels, the image sensor was 4/3 CMOS, the lens equivalent focal length was 24 mm, and the aperture was f/2.8-f/11. The images were collected at 10:00 a.m. on November 1 and November 2, 2023. The weather was clear and cloudless, and the images were taken obliquely. The flight speed was 3 M/s, the flight time was 25 min, the heading overlap was 80%, and the lateral overlap was 70%. The visible light images at the seedling stage were taken at a height of 3 m, with a resolution of 4000 × 2250 pixels, and a total of 2000 images were taken, some images of wheat seedling were shown in **Figure 2**. In order to improve the efficiency of data processing and facilitate the training of neural network, the original image is cut into an image with a resolution of 512 × 512 pixel, as shown in **Figure 2a**. Use the annotation tool to annotate the self-built wheat seedling dataset image (Wang et al., 2021; Zhu et al., 2022), as shown in **Figure 2b**.

### Data processing

Wheat seedling is small and densely distributed, which is prone to occlusion and overlap, resulting in missed detection, making the method based on box annotation difficult to be effectively applied. Therefore, we adopt a low-cost, convenient and fast point annotation method. The point annotation represents the position coordinates of wheat seedling in the image. This method can not only mark the image into block, but also support the random scaling of the marked area. For the areas with dense distribution of wheat seedling, serious occlusion and overlap in the image, we use the method of point annotation after magnification, which effectively improves the annotation speed and quality. The labeling points are selected at the root and stem of wheat seedlings with obvious characteristics, which is convenient for the subsequent training of target detection network.

### Data expansion

This method strengthens the generalization ability of the model by means of data expansion, thus successfully avoiding the possible over fitting problem of the model. The specific data enhancement operation is realized by image flipping, image rotation, brightness balance and adding gaussian noise. A single image typically contains a vast amount of data, and through data processing, it can yield over 100 pieces of useful information. During drone flights, factors such as low altitudes and slow speeds lead to variations in shooting angles and lighting within the same area. Additionally, images taken from different flight paths of the same location may exhibit differences. This diverse data enhances the robustness of deep neural networks, preventing overfitting to specific data, and thus holds significant value in training deep neural networks. After data enhancement, 2000 original images were processed to obtain a total of 4000 wheat seedling images and their corresponding annotation points, which together constitute the wheat seedling image dataset, and meet the training requirements of deep learning. According to the ratio of 7:2:1, the wheat seedling image dataset is divided into training set, validation set and test set. The training set, validation set and test set include 2800, 800 and 400 wheat seedling images respectively.

### Deep learning model

**Figure 3:**
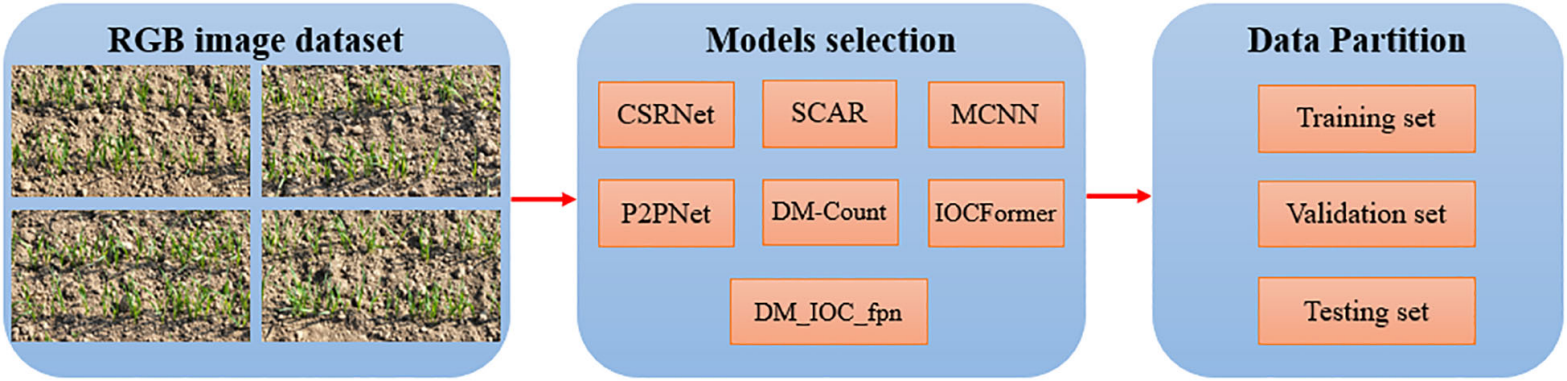
Framework of wheat seedling number detection using deep learning model.

The deep learning model is an important part of the field of artificial intelligence. It’s basic principle is built by the convolution layer, pooling layer and full connection layer, which is used for automatic feature extraction and classification of input data (Shafiq and Gu, 2022). Convolution layer is the core layer of deep learning model to extract data feature such as image, which is mainly used to automatically extract local features of data. The pool layer is located behind the convolution layer, and it’s main function is to reduce the dimension of the feature map output from the convolution layer, which can’t only significantly reduce the amount of data, but also reduce the complexity of subsequent calculation. The full connection layer integrates the feature extracted from the previous convolution layer and the pool layer. Each neuron is connected to all neurons in the previous layer. By weighted summation of the input feature, the final prediction result is output. The convolution layer, pooling layer and full connection layer work together to build a powerful deep learning model architecture. In this study, we carefully selected seven most advanced deep learning models, namely CSRNet, SCAR, MCNN, P2PNet, DM-Count, IOCFormer and DM_IOC_fpn. These models showed unique advantages and excellent performance in the field of deep learning, and provided a solid technical foundation for subsequent research and analysis. Using the transfer learning method, all models have completed the pre training using the transfer learning method. **Figure 3** shows the schematic diagram of data set, model selection and data set division.

### DM_IOC_fpn architecture

**Figure 4:**
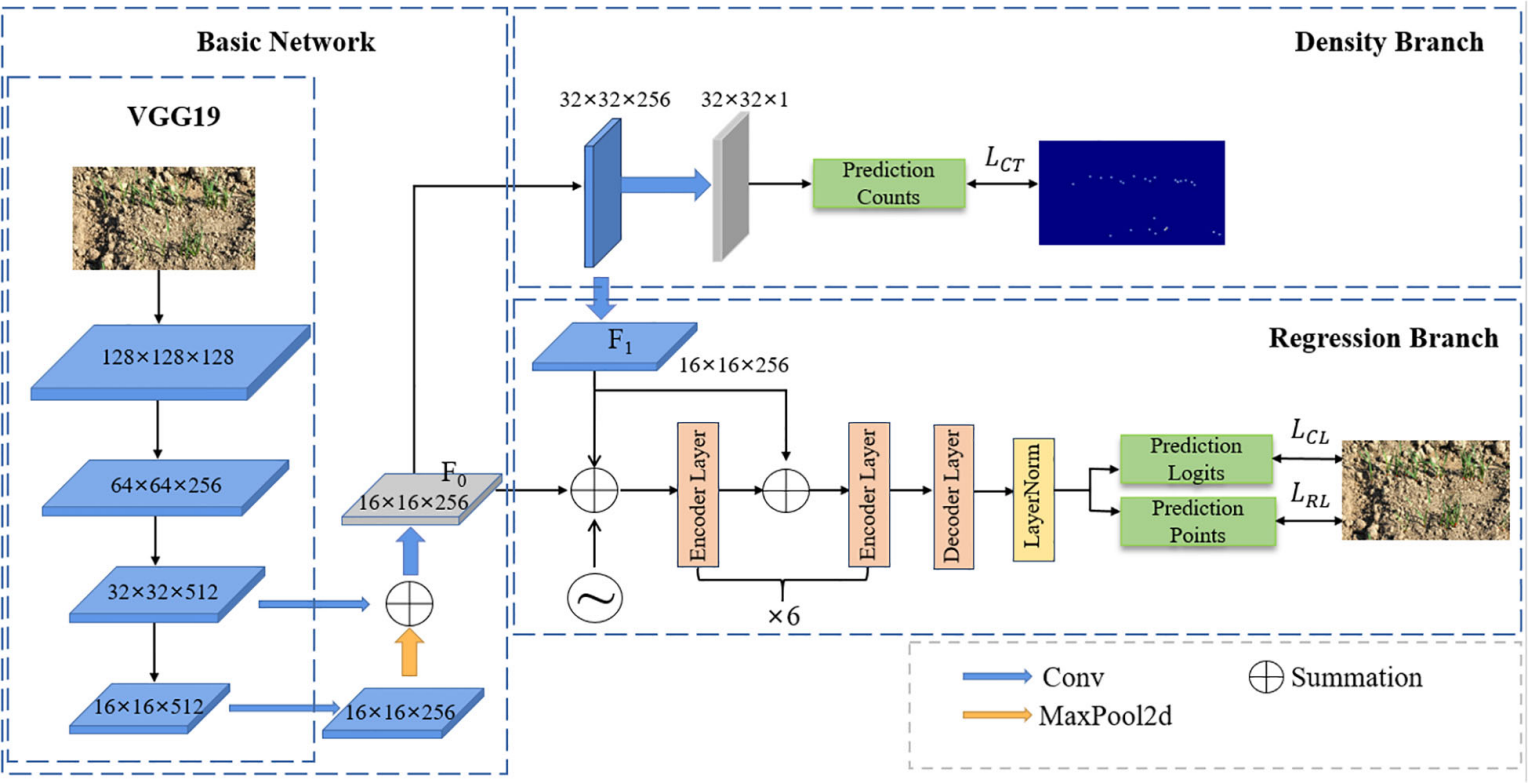
DM_IOC_fpn architecture.

In this study, VGG19 is used as the backbone network to creatively build a dual branch structure, which is density branch and regression branch respectively. The network architecture of DM_IOC_fpn is shown in **Figure 4**. The wheat seedling image is input into the model. Firstly, through the backbone network VGG19, the feature maps of the 12th and 16th layers are extracted and input into the feature pyramid network. The feature pyramid network fuses the low-level feature high-resolution and high-level high semantic information to obtain the feature map $F_{0}$ , with a feature dimension of 16 × 16 × 256. The feature map $F_{0}$ output from the feature pyramid is input into the density branch and the regression branch respectively. First, it enters the density branch, and generates the density sensing feature map $F_{1}$ through the convolution layer, with a feature dimension of 16 × 16 × 256. Then, the feature map $F_{0}$ and $F_{1}$ are added and input into the encoder, so that the regression branch to not only rely on local appearance features when predicting the position of wheat seedlings, but also perceive global density distribution context information. When wheat seedlings are occluded or overlap, the model can infer the potential presence of occluded areas based on the distribution pattern of surrounding wheat seedlings, thereby significantly reducing missed detections. Finally, the features output by the decoder are processed by a classification head and a regression head to generate predicted segmentation maps and coordinate information, respectively, completing accurate localization and counting of wheat seedlings.

### Density enhancement encoder module

**Figure 5:**
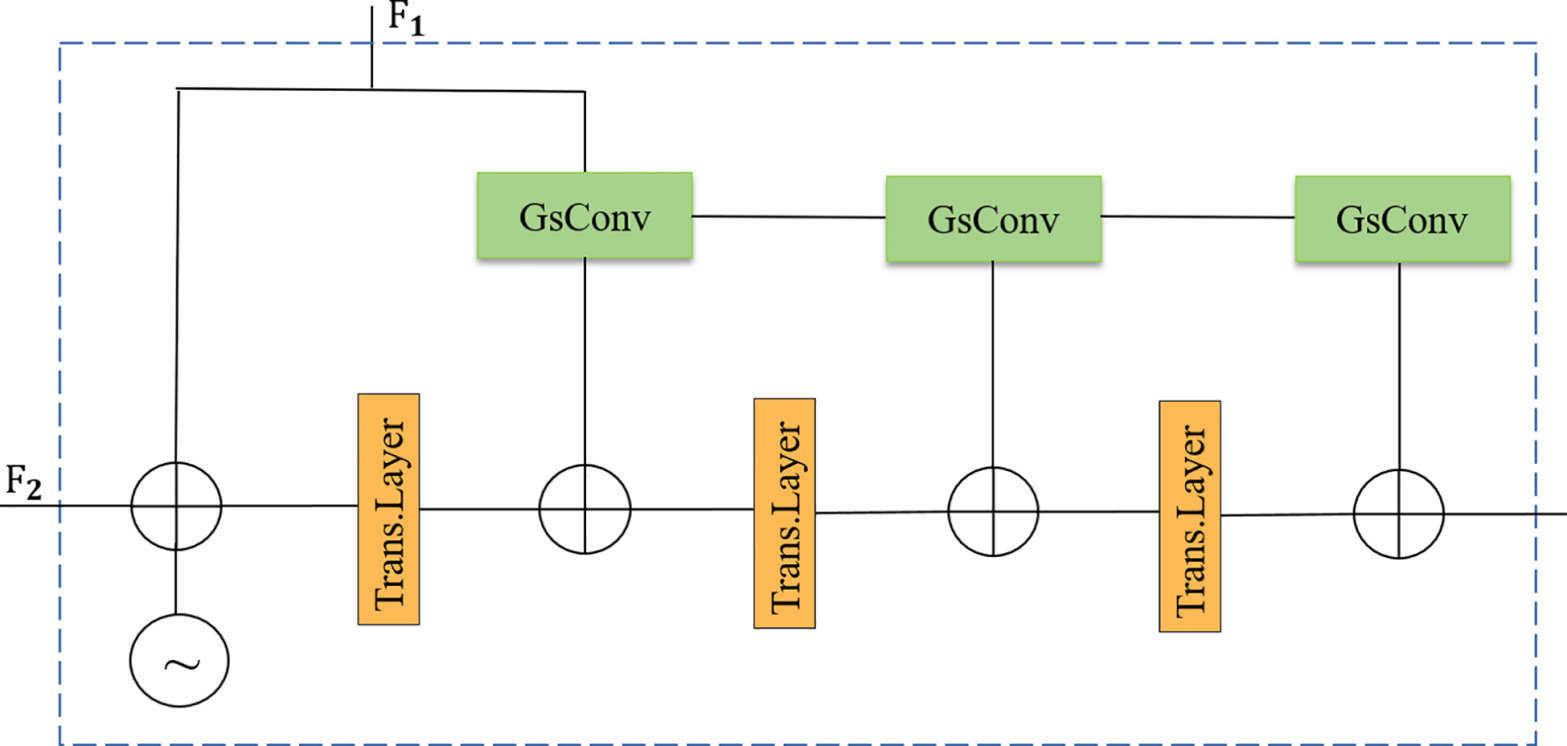
Density-enhanced encoder module.

**Figure 6:**
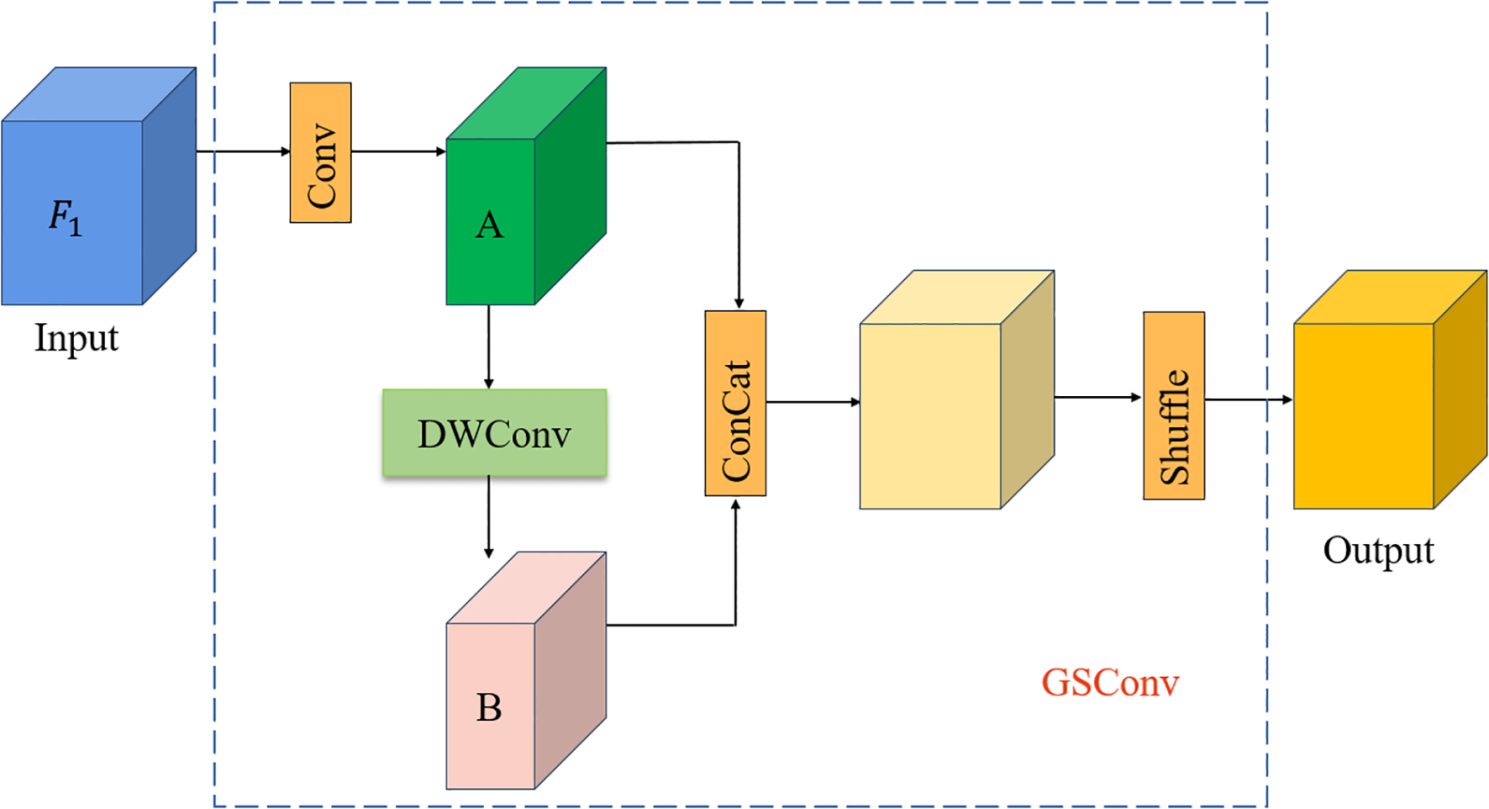
GSConv module.

This research improves the density enhancement encoder module in IOCFormer, as shown in **Figure 5**. Replace Convs with GSConv to make it lighter, compared with Convs, GSConv divides the traditional convolution process into two steps: depthwise convolution and pointwise convolution, and adopts a strategy of channel halving and then concatenation, significantly reducing the number of parameter in the model at the structural level, as shown in **Figure 6**. The density sensing feature $F_{1}$ from the density branch is input into GSConv. First, the standard convolution is performed to generate the feature map A, the number of channels becomes half of the output feature map, and then the depth convolution is performed to generate the feature map B. The feature map A and B are spliced in the channel dimension, which greatly enriches the diversity of features, enables the model to learn more robust feature representations from different receptive fields and computational paths. Then, a shuffle operation is performed, which is crucial for solving the occlusion problem. It effectively avoids the isolation of feature information in the channel dimension, prompting the model to integrate local details (such as partially visible wheat leaf tips) with surrounding contextual information, thus making more accurate judgments on partially occluded or heavily overlapped wheat seedlings, and improving the model’s generalization ability.

### Loss function

In order to improve the convergence speed, accuracy and generalization ability of the model, the model uses three different loss functions, namely counting loss, classification loss and regression loss.

Count loss is calculated by calculating the absolute value of the difference between the predicted number of wheat seedlings and the real number of wheat seedlings in each image, and then added and divided by the total sample. It is defined as follows:


$$L_{CT}=\frac{1}{N}\sum_{i=1}^{N}\left|C_{i}^{pred}-C_{i}^{GT}\right|$$

Where N is the total sample, and $C_{i}^{pred}$ and $C_{i}^{GT}$ are the predicted value and the real value of a sample respectively. The binary cross entropy loss function is used for classification loss, in areas where wheat seedlings are densely overlapped, the appearance characteristics are blurred, which can easily lead to misjudgment. The classification loss is achieved through a binary discrimination mechanism, which forces the model to learn to distinguish the subtle differences in characteristics between wheat seedlings and non-wheat seedlings (such as soil, shadows, or overlapping edges). This can effectively reduce false positives or missed detections caused by overlap.it is defined as follows:


$$L_{CL}=-\left[ylog\left(a\right)+\left(1-y\right)\log\left(1-a\right)\right]$$

Where y is the real label, value is 0 or 1, a is the output of the model, and the prediction is the probability of wheat seedlings.

Use regression loss to calculate the difference between the predicted coordinates and the real coordinates, and supervise the learning of the model. It is defined as follows:


$$L_{RL}=\frac{1}{M}\sum_{i=1}^{M}\left|Y_{i}^{pred}-Y_{i}^{GT}\right|$$

Where M is the total number of wheat seedlings, $y_{i}^{GT}$ is the real coordinate, and $y_{i}^{pred}$ is the prediction coordinate. The total loss function of the model is: $L=\alpha L_{CT}+L_{CL}+L_{RL}$ Where α is a super parameter and is set to 0.5, so as to balance the contribution of positive and negative samples.

### Network training

This paper uses Ubuntu operating system and NVIDIA GeForce RTX3090 as GPU, which is implemented based on PyTorch, CUDA and Python. The batch size is limited by the data size and GPU memory. The batch size was tested in the range of 8~32, and the results showed no significant difference. Therefore, 8 was selected as the optimal value that balances efficiency and stability. The learning rate was selected based on grid search using the validation set. The optimal initial value was determined through cross-validation, and the learning rate decay strategy was used to accelerate convergence. Therefore, the learning rate was set to 0.001. The training rounds were 1000, and early stopping was used to prevent overfitting during training. The model usually converges after hundreds of rounds. Adam was selected as the optimizer, which showed good convergence performance in the experiment.

### Evaluation index

In this study, RMSE, MAE and R2 were used to evaluate the detection effect of wheat seedling, evaluate model performance using GFLOP, parameter, memory, and time consumption. The calculation formulas of RMSE, MAE and R2 are as follows:


$$RMSE=\sqrt{\frac{1}{N}\sum_{i=1}^{N}{\left(p_{i}-q_{i}\right)}^{2}}$$

$$MAE=\frac{1}{N}\sum_{i=1}^{N}\left|p_{i}-q_{i}\right|$$

$$R^{2}=1-\frac{\sum_{i}{\left(p_{i}-q_{i}\right)}^{2}}{\sum_{i}{\left(\bar{p}-q_{i}\right)}^{2}}$$

Where N is the number of images, Pi is the actual number of the ith image, and qi is the predicted number of the ith image.

## Results and analysis

### Training loss results of different deep learning models

**Figure 7:**
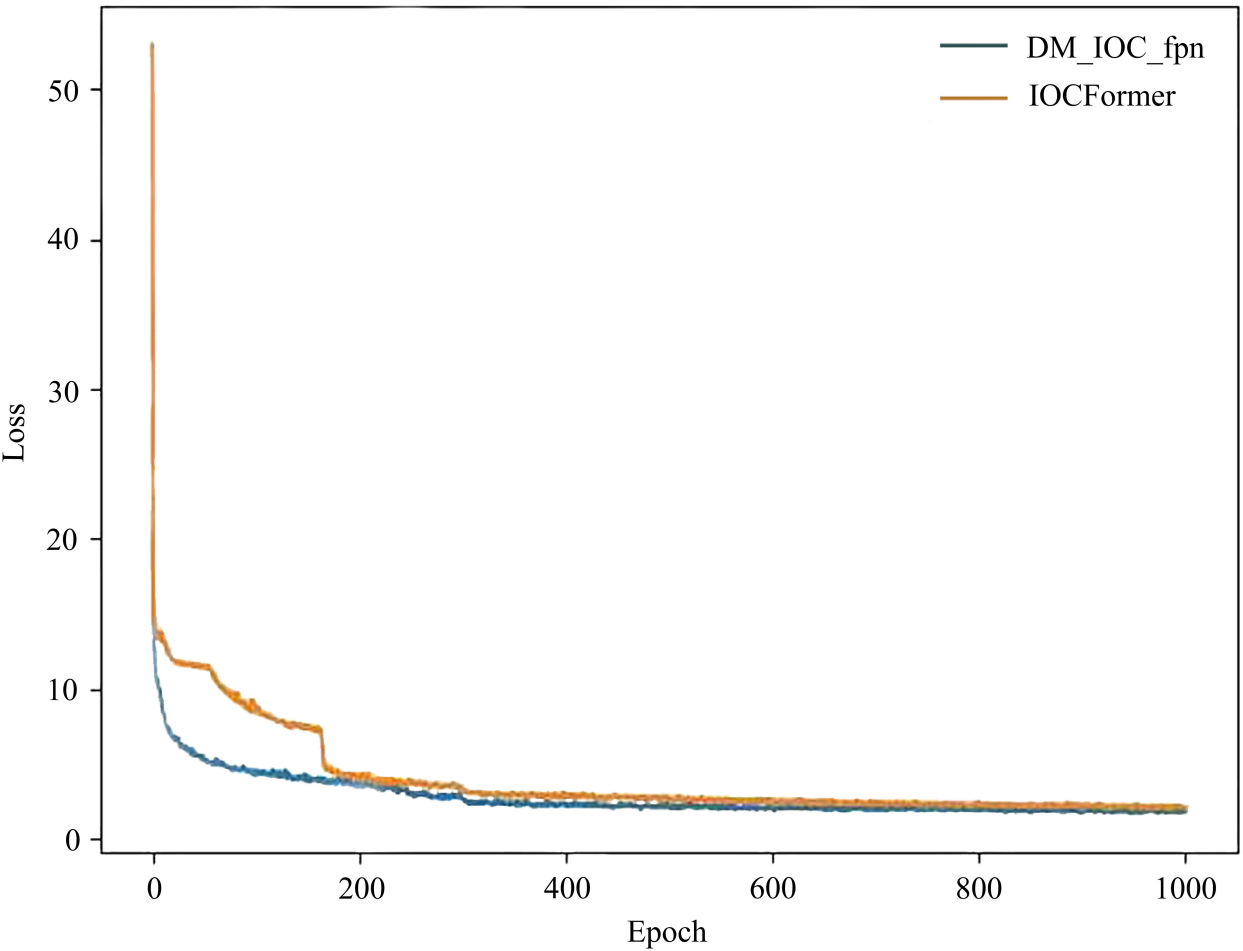
Training loss results of IOCFormer and DM_IOC_fpn.

To verify the superiority of DM_IOC_fpn, the training loss results of IOCFormer and DM_IOC_fpn are shown in **Figure 7**, where the horizontal axis represents the number of training times and the vertical axis represents the loss value of each round. As increasing the iterations number, IOCFormer and DM_IOC_fpn converge and remain stable before 400 epochs, the training loss gradually decreases, then converges and remains stable, the loss value of IOCFormer is higher than that of DM_IOC_fpn. At the beginning of the network training iteration, the training loss curve of DM_IOC_fpn decreases rapidly. At the middle of the training iteration, the training loss curve of DM_IOC_fpn decreases moderately. At the end of the training iteration, the training loss curve of DM_IOC_fpn remains stable, indicating that DM_IOC_fpn has excellent stability.

### Comparative analysis of wheat seedling detection results using different deep learning models

In order to compare the detection effect of DM_IOC_fpn model and the current mainstream target detection model on wheat seedlings in the field, CSRNet, SCAR, MCNN, P2PNet, DM-Count, IOCFormer and DM_IOC_fpn were selected for comparative analysis. **Table 1** shows the comparison results of evaluation indexes of different models on wheat seedling data sets. The RMSE of CSRNet, SCAR, MCNN, P2PNet, DM-Count, IOCFormer and DM_IOC_fpn models were 7.49, 3.36, 14.62, 6.17, 3.10, 4.69 and 2.91, respectively. The RMSE of MCNN was the largest and that of DM_IOC_fpn was the smallest. The MAE of CSRNET, SCAR, MCNN, P2PNet, DM-Count, IOCFormer and DM_IOC_fpn models were 6.10, 2.83, 13.72, 4.83, 2.38, 3.27 and 2.23, respectively. The RMSE of MCNN was the largest and that of DM_IOC_fpn was the smallest. The DM_IOC_fpn model proposed has the highest accuracy in counting wheat seedling, with RMSE of 2.91 and MAE of 2.23, which are reduced by 0.19 and 0.15 respectively compared with DM-Count. Therefore, in higher image resolution and more complex environment, our proposed DM_IOC_fpn still shows excellent performance, and the two kinds of counting errors are also the smallest, which improves the accuracy of wheat seedling counting, and has practical value for wheat seedling counting.

**Table 1:** Comparison analysis of experimental results of different deep learning models on the wheat seedling dataset.

Models	RMSE	MAE
CSRNet	7.49	6.10
SCAR	3.36	2.83
MCNN	14.62	13.72
P2PNet	6.17	4.83
DM-Count	3.10	2.38
IOCFormer	4.69	3.27
DM_IOC_fpn	2.91	2.23

### Comparison of computation, parameter count, memory and time per image for different deep learning models

This study evaluated the possibility of implementing different deep learning models on drone platforms. We compared the performance of seven mainstream detection models in terms of computation, parameter count, memory, and time per image, as shown in **Table 2**. MCNN has the lowest FLOPs, parameter count, memory, and time per image, but its counting accuracy is relatively low. DM_IOC_fpn has a computation of 22.99 FLOPs, which is between MCNN and IOCFormer, reflecting a balanced design between computational efficiency and detection performance. DM_IOC_fpn contains 87.26 M parameters and has a relatively complex architectural design. Compared with the lightweight model MCNN (0.51M), the parameter count has increased significantly, but this provides the model with stronger feature expression capabilities. DM_IOC_fpn has a memory of 7.15 GB, which is relatively high among the compared models, but still within the acceptable range of GPUs. DM_IOC_fpn provides an inference speed suitable for drone deployment while maintaining optimal detection accuracy, analyzing 10 high-resolution wheat seedling images per second. Therefore, DM_IOC_fpn achieves a better balance between accuracy and efficiency, providing a feasible solution for real-time wheat seedling detection in the field.

**Table 2:** Comparison of computation, parameter count, memory, and time per image for different deep learning models.

Models	GFLOP (G)	Parameters (M)	Memory (G)	Time (s/image)
CSRNet	20.72	16.26	5.15	0.09
SCAR	20.74	16.29	5.18	0.10
MCNN	1.34	0.13	0.12	0.04
P2PNet	20.04	19.21	6.65	1.72
DM-Count	20.66	20.50	4.28	0.06
IOCFormer	32.26	51.65	6.62	0.24
DM_IOC_fpn	22.99	25.78	7.15	0.11

### The relationship between the actual and predicted values of wheat seedling counting using different deep learning models

**Figure 8:**
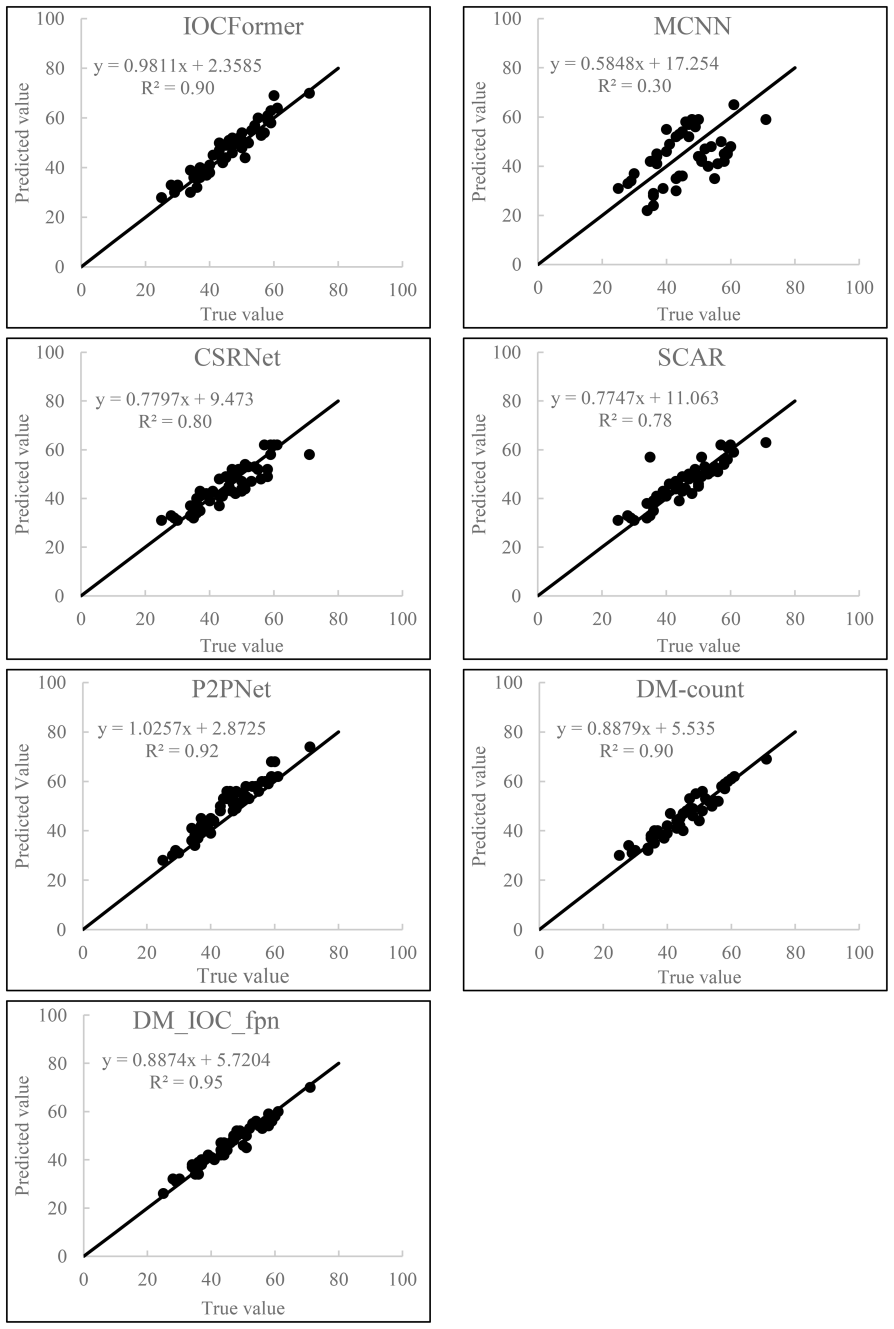
Relationship between actual and predicted values of wheat seedling counting using different deep learning models.

In order to verify the counting effect of the detection model proposed on the self-built dataset, the counting experiment was carried out on 50 wheat seedling images in the self-built dataset, and the relationship between the actual values and the predicted values of the test data is shown in **Figure 8**. It can be seen from **Figure 8** that the predicted value and the real value of the wheat seedling dataset from the perspective of UAV have a high correlation, indicating that the DM_IOC_fpn model proposed by us not only has high counting accuracy, but also has good generalization ability. R^2^ varies across different deep learning models, R^2^ of IOCFormer, MCNN, CSRNET, SCAR, P2PNet, DM-Count and DM_IOC_fpn models are 0.90, 0.30, 0.80, 0.78, 0.92, 0.90 and 0.95 respectively, The R^2^ of the DM_IOC_fpn model is the highest, while the R^2^ of the MCNN is the lowest. Compared with IOCFormer model, the R^2^ of DM_IOC_fpn model increased by 5%. In conclusion, the DM_IOC_fpn model proposed has good robustness and can accurately detect and count wheat seedling.

### Analysis of typical wheat seedling images in the testset

**Figure 9:**
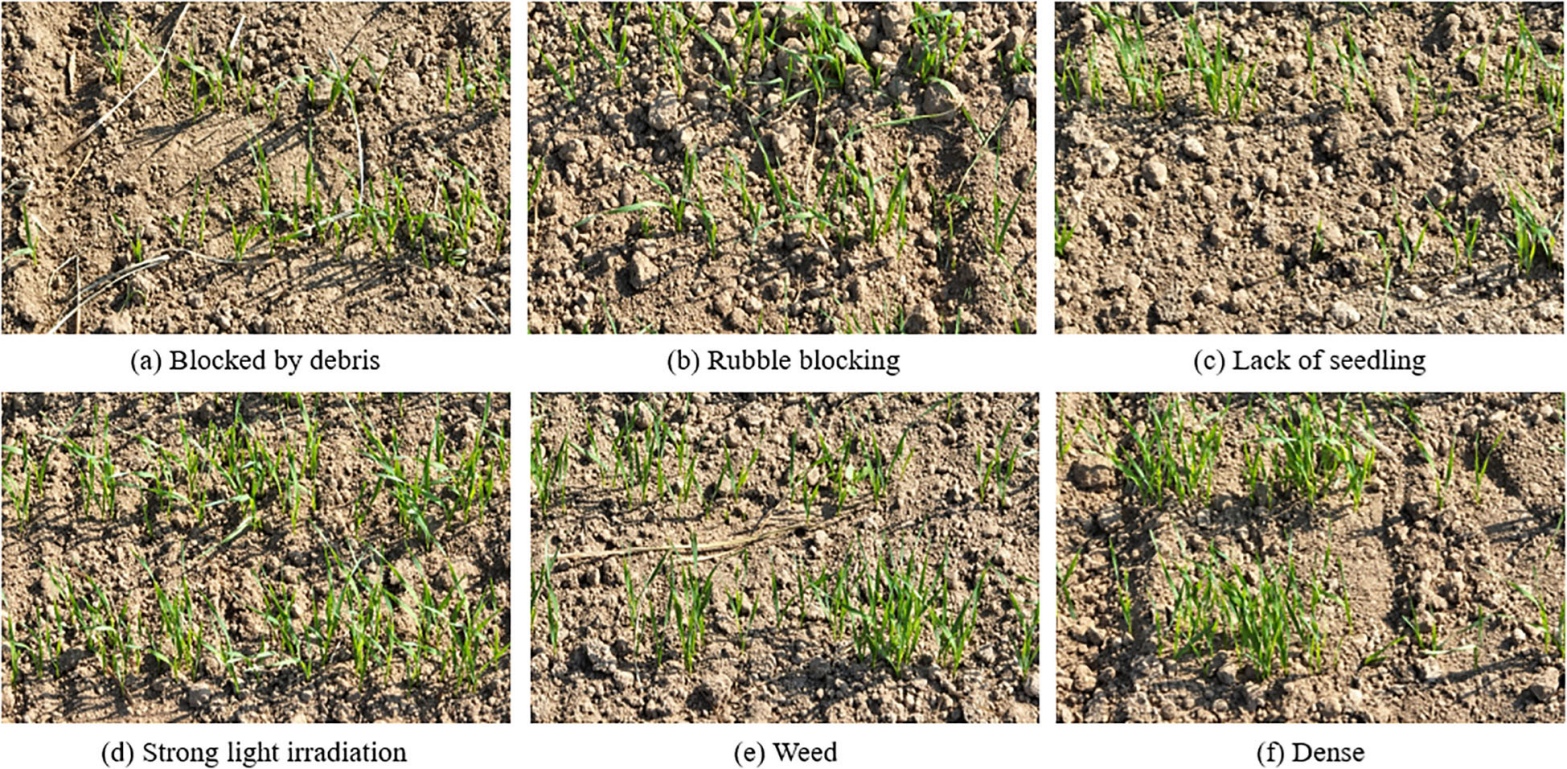
Typical image example of the testset.

Affected by factors such as imaging angle, sundries and weeds, and wheat seedling density, there are various typical challenge scenarios in the testset, **Figure 9** shows six typical image examples. **Figure 9a** shows that there are sundries between wheat seedlings; **Figure 9b** shows the presence of debris between wheat seedlings; **Figure 9c** shows the uneven growth state of wheat seedlings and the phenomenon of lacking seedlings; **Figure 9d** shows the image of wheat seedlings taken during the day and exposed to strong sunlight; **Figure 9e** shows the presence of weed shelter between wheat seedlings; **Figure 9f** some wheat seedlings grow too densely, crowded and disorderly. The aforementioned complex external environmental conditions have heightened the difficulty of wheat seedling detection, leading to instances of missed or false detections when using other target detection models. This underscores the exceptional robustness of our research model in addressing such challenges.

### Visualization of wheat seedling counting using different deep learning models

**Figure 10:**
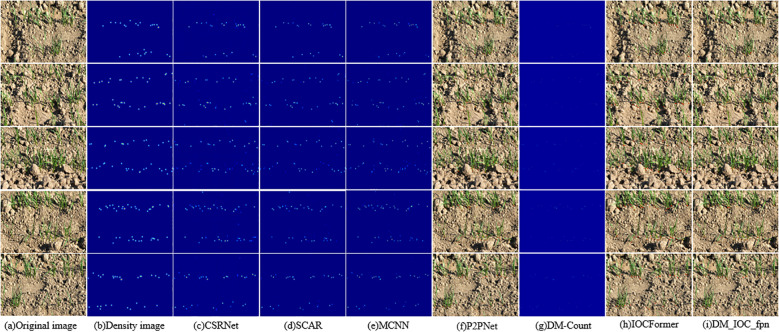
Visualization of wheat seedling counting results using different deep learning models.

**Figure 10** shows the visualization results of wheat seedling counting on the self-built dataset of different deep learning models, **Figure 10a** is the original image, and **Figure 10b** is the density map generated by point annotation, which serves as the true value for the density map counting method, **Figures 10c-i** present the counting results forCSRNET, SCAR, MCNN, P2PNet, DM-Count, IOCFormer and DM_IOC_fpn, respectively. Visualize the data through a density plot, the darker the color of density map, the more wheat seedlings. The counting accuracy of these density map-based methods is not high, and the generated density maps cannot directly identify the location of wheat seedlings, thus failing to provide more supporting information for downstream tasks. **Figures 10f, h, i** show the prediction results of P2PNet, IOCFormer, and DM_IOC_fpn, respectively. These results are more intuitive wheat seedling coordinates. Due to the introduction of dual branches in DM_IOC_fpn, which enhances the local and global contextual feature information of wheat seedlings, its prediction values are closer to the true values when counting wheat seedling images affected by factors such as occlusion, overlap, and illumination, resulting in smaller counting errors. The DM_IOC_fpn model proposed can fully extract the image global feature, and the comprehensive detection effect is the best. **Figure 10** shows the visualization results of wheat seedling detection by seven detection networks. When the counting of wheat seedling images is affected by factors such as occlusion and overlap, the convolutional neural network has missed detection. DM_IOC_fpn can accurately locate and recognize wheat seedling targets, and can eliminate the detection errors in the original model. It not only improves the detection accuracy of wheat seedlings, but also reduces the misjudgment of wheat seedlings, showing better generalization performance.

### Ablation test

In order to verify the effectiveness of the improved model, ablation experiments were carried out in combination with the test set, and the effectiveness of the improved DM_IOC_fpn model was verified based on the IOCFormer model. It can be seen from **Table 3** that adding density branches, RMSE and MAE decreased by 1.16 and 0.72 respectively compared with IOCFormer model, indicating that density branches can improve the ability of the model to capture the characteristics of wheat seedlings. Compared with IOCFormer model, RMSE and MAE were increased by 0.48 and 0.63 respectively by introducing regression branch, indicating that regression branch reduced the accuracy of wheat seedling detection. When adding density branch and regression branch, DM_IOC_fpn model performs best in comprehensive performance. Compared with IOCFormer model, RMSE and MAE are reduced by 1.78 and 1.04 respectively. The comprehensive ablation test proves the effectiveness of the improved DM_IOC_fpn model.

**Table 3:** Ablation test results.

Baseline network	Regression branch	Density branch	RMSE	MAE
IOCFormer			4.69	3.27
		✓	3.53	2.55
	✓		5.17	3.90
	✓	✓	2.91	2.23

### Verification of test results

In order to evaluate the automatic counting performance of DM_IOC_fpn model in the field environment, 20 wheat seedling images were randomly selected from the testset, and the accuracy was evaluated by manually marking the true value and the predicted value of the model. See **Table 4** for wheat seedling counting results of different models. The average relative error of DM_IOC_fpn counting is 4.53%, which is 3.15%, 17.06%, 3.71%, 1.81%, 5.98% and 0.96% lower than that of IOCFormer, MCNN, CSRNET, SCAR, P2PNet and DM-Count, indicating that the relative error of DM_IOC_fpn is more concentrated and the result of wheat spike seedling is more stable.

**Table 4:** Counting results of wheat seedlings in different models.

Image number	True value	IOCFormer	Relative error (%)	MCNN	Relative error (%)	CSRNet	Relative error (%)	SCAR	Relative error (%)	P2PNet	Relative error (%)	DM-count	Relative error (%)	DM_IOC_fpn	Relative error (%)
1	44	49	11.36	36	18.18	41	6.81	39	11.36	53	17.70	42	4.54	47	6.81
2	58	59	1.72	42	27.58	52	10.34	56	3.44	60	3.44	59	1.72	54	6.89
3	48	50	4.16	59	22.91	42	12.50	42	12.50	49	2.08	46	4.16	52	8.33
4	55	60	9.09	35	36.36	52	5.45	53	3.63	56	1.81	52	5.45	54	1.81
5	50	54	8.00	59	18.00	47	6.00	48	4.00	55	10.00	44	12.00	51	2.00
6	34	39	14.70	22	35.29	37	8.82	38	11.76	41	20.58	33	2.94	38	11.76
7	59	63	6.77	45	23.72	62	5.08	61	3.38	68	15.25	60	1.69	56	5.08
8	47	52	10.63	57	10.63	43	8.51	48	2.12	51	8.51	49	4.25	48	2.12
9	60	69	15.00	48	20.00	62	3.33	62	3.33	68	13.33	61	1.66	58	3.33
10	40	38	5.00	55	37.5	42	5.00	41	2.50	39	2.50	42	5.00	41	2.50
11	37	40	8.10	45	21.62	43	16.21	40	8.10	45	21.62	39	5.40	38	2.70
12	43	45	4.65	30	30.23	41	4.65	45	4.65	48	11.62	44	2.32	44	2.32
13	51	50	1.96	42	17.64	54	5.88	57	11.76	58	13.72	56	13.72	50	1.96
14	50	51	2.00	44	12.00	44	12.00	45	10.00	52	4.00	44	12.00	51	2.00
15	45	44	2.22	54	20.00	43	4.44	43	4.44	53	17.77	40	11.11	44	2.22
16	54	57	3.70	48	11.11	53	1.85	51	5.55	58	7.40	50	7.40	56	3.70

### Generalization of different deep learning models in wheat ear detection

**Figure 11:**
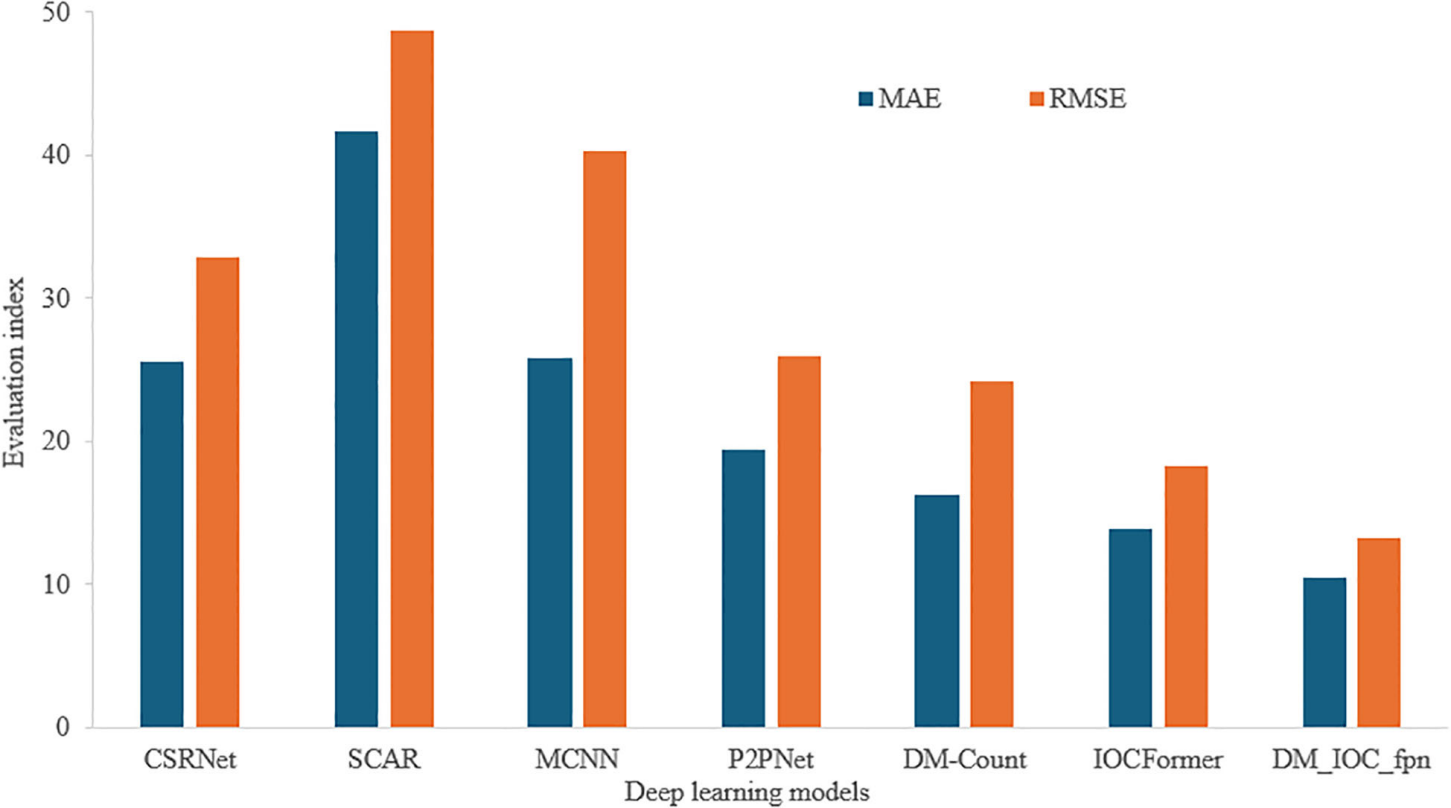
Comparison of experimental results of different deep learning models on wheat spike dataset.

In order to verify the effectiveness of DM_IOC_fpn model in wheat ear detection task, we used the common dataset wheat ear detection dataset (WEDD), which contains 236 high-resolution images with a resolution of 6000 × 4000. In this study, the WEDD test is zero-shot, the model trained on the self-built wheat seedlings dataset, which was tested on the WEDD. **Figure 11** shows the experimental results of different deep learning models on the wheat ear dataset, SCAR model has the largest MAE and RMSE, while DM_IOC_fpn model has the smallest MAE and RMSE. The DM_IOC_fpn we proposed still maintains the optimal performance, with MAE of 10.46 and RMSE of 13.28. Compared with IOCFormer, the MAE and RMSE of DM_IOC_fpn decreased by 3.41 and 4.98, respectively. Compared with other detection models, the counting error of DM_IOC_fpn is the smallest. Therefore, the method proposed in this study can accurately detect the small target number of wheat ear, and better solve the technical problems of occlusion and overlapping of the number of wheat ear, thereby improving the accuracy of wheat ear counting.

### Five-fold cross-validation of different deep learning models

To prevent model overfitting and comprehensively evaluate its generalization ability, five-fold cross-validation was selected to train and validate different data subsets. Five-fold cross-validation ensures that the proportion of samples in each fold class is exactly the same as the category proportion in the original dataset during partitioning. The self-built wheat seedling dataset was randomly divided into five subsets of similar size, with four of them selected as the training set and the remaining one as the validation set. Random seeds are fixed, and their core role in cross validation is to ensure that the data partitioning results are reproducible, using the same augmentation between folds for training. The results of the five-fold cross-validation are shown in **Table 5**. The RMSE of the IOCFormer model ranged from 4.69 to 5.34, and the MAE ranged from 3.27 to 3.75. The paired t-test results showed that the differences between other models and the IOCFormer model reached significant or extremely significant levels, with the DM_IOC_fpn model showing an extremely significant difference from the IOCFormer model. Despite statistically significant differences, the numerical results showed that the DM_IOC_fpn model maintained a relatively stable leading performance, with RMSE ranging from 2.91 to 3.70 and MAE ranging from 2.23 to 2.51, outperforming other models. The differences between the CSRNet, MCNN, P2PNet, IOCFormer models and the DM_IOC_fpn model reached extremely significant levels. In summary, the DM_IOC_fpn model had the lowest RMSE and MAE, indicating that it performed best in terms of accuracy and stability, highlighting the effectiveness of its structure.

**Table 5:** Five-fold cross validation results of different deep learning models.

Models	Index	One fold	Two fold	Three fold	Four fold	Five fold	t-test	t-test
IOCFormer	RMSE	4.69	5.18	4.79	5.10	5.34	23.25**	37.12**
	MAE	3.27	3.69	3.40	3.71	3.75	17.69**	24.40**
CSRNet	RMSE	7.49	7.82	8.18	8.13	7.92	-8.94**	1.73
	MAE	6.10	6.55	7.10	6.89	6.48	-3.93*	4.87**
SCAR	RMSE	3.36	3.45	3.25	4.12	3.79	29.79**	34.97**
	MAE	2.83	2.95	2.79	3.51	3.15	41.75**	46.46**
MCNN	RMSE	14.62	15.32	13.81	15.71	14.58	8.35**	17.86**
	MAE	13.72	14.25	13.15	14.51	13.52	12.30**	20.94**
P2PNet	RMSE	6.17	6.28	6.57	6.37	7.18	-9.97**	2.00
	MAE	4.83	4.96	5.36	5.44	5.49	-5.98**	1.63
DM-Count	RMSE	3.10	3.91	3.51	3.81	3.68	/	13.99**
	MAE	2.38	3.22	2.44	2.51	2.71	/	12.06**
DM_IOC_fpn	RMSE	2.91	3.15	3.37	3.70	3.46	-12.63**	/
	MAE	2.23	2.48	2.39	2.41	2.51	-23.80**	/

## Discussion

As one of the important characteristic parameters of wheat, plant number is of great significance for wheat breeding and yield estimation. Deep learning target detection technology is based on deep convolutional neural network, which can automatically extract features from the input image data, and then identify and locate specific target objects (Zhang et al., 2024). Based on the massive dataset with different light intensity and hue changes, diverse shooting angles and various complex backgrounds, the deep convolutional neural network model is systematically and carefully trained, so as to achieve high-precision detection of the target object. Common target detection algorithms include Faster R-CNN (Li et al., 2022), YOLO (Joseph and Ali, 2016) and SSD, MCNN, CSRNet, SCAR and DM-Count. At present, the deep learning target detection technology is developing rapidly. With its significant advantages in image detection, it is widely used in corn seedling, wheat spike (Khaki et al., 2022), rice (Wu et al., 2019), sugar beet (Barreto et al., 2021), rape (Li et al., 2023), peanut (Lin et al., 2022) and so on to carry out the exploration of target detection and counting. In addition, researchers have proposed a deep learning network structure to achieve crop counting task in natural scene. However, the existing deep learning methods mainly focus on counting, and there are many other applications that have not been mined (Liu et al., 2020; Wang et al., 2022). Although deep learning has made significant progress in image counting tasks, complex background interference and noise pollution are still technical bottlenecks to be solved, which have been the key problems restricting the improvement of counting accuracy.

In order to further verify the feasibility of this method in the aspects of small wheat seedling, dense distribution, mutual shielding and serious overlapping, this method was compared with several schemes to solve the above problems. Jin et al. (2017) used the support vector machine method to estimate the wheat plant density based on the high-resolution image taken by UAV, and the relative RMSE was 14.31%. However, due to the small sample size of the dataset, the experimental results were easy to overfit. Bai et al. (2023) can accurately and effectively estimate the number of rice plants using RiceNet network, with MAE and RMSE of 8.6 and 11.2 respectively; However, their method reduces the counting performance of rice due to the emergence of weed. Zang et al. (2024) proposed a DMseg-Count model to realize the automatic detection and counting of wheat ears; due to the limited storage space of the mobile phone, it may not be able to continuously collect data for a long time, resulting in incomplete data. In addition, if the mobile phone runs out of power, insufficient storage space and other unexpected circumstances occur during the collection process, it will also cause data loss or interruption, affecting the integrity of the data set. Liu et al. (2017) developed an automatic measurement method of wheat density based on machine vision, and the average relative error of density estimation is 12%, this method needs to recalibrate the model at each new experimental site. Lu and Cao (2020) proposed a new network TasselNetV3 for rice plant counting, which is suitable for counting on rice plant images taken by fixed cameras, and can better solve the problem of mesoscale distortion in plant images.

In recent years, significant progress has been made in wheat seedling detection and counting technology, which has attracted extensive attention of researchers. However, wheat growth is a complex and dynamic process, especially reflected in the color characteristics and background changes of wheat seedlings. The existing target detection models are usually optimized for specific growth stages when they are designed and applied, but their adaptability to other growth stages or field scenes is often limited, and the detection effect of small targets and dense targets is poor. The wheat seedling dataset captured by UAV in this study has a wider perspective than the WEDD. Because the detection accuracy of the wheat seedling dataset captured by UAV in this paper is higher than the WEDD, it further verifies that DM_IOC_fpn has strong wheat seedling detection ability and generalization ability in different perspectives. When agricultural experts manually count wheat seedlings, in the face of difficult samples that are difficult to determine, they usually judge that wheat seedlings belong to single or multiple plants according to the local characteristics of the roots and stems of wheat seedlings and the overall development of leaves. Inspired by this, this study proposes a wheat seedling number detection model DM_IOC_fpn, which can accurately detect the number of small target wheat seedlings, and better solve the problem of occlusion and overlapping of wheat seedling.

The innovativeness of the DM_IOC_fpn model lies in its achievement of model combination to architecture fusion for agricultural dense target scenarios. The IOCFormer model excels in instance localization but has weaker global density perception; whereas the DM-Count model excels in density estimation but lacks precise localization capabilities. The advantages of the DM_IOC_fpn model are reflected in the significant performance improvement and robustness enhancement brought by its overall architecture. This study improves the existing target detection model IOCFormer and proposes the DM_IOC_fpn model for wheat seedling count detection, which integrates local and global features. The model builds a dual-branch structure based on IOCFormer to obtain local and global contextual feature information of wheat seedlings. It introduces a density-enhanced encoder module to enhance the model’s feature extraction ability. The total loss function is constructed by combining counting loss, classification loss, and regression loss, reducing the technical challenges of false positives and false negatives in wheat seedling detection. From the above experimental results, it can be seen that compared with other mainstream counting models, the DM_IOC_fpn model proposed in this study achieves a better balance between counting accuracy and efficiency, providing a feasible solution for real-time wheat seedling detection in the field. The model builds a dual-branch structure based on IOCFormer to obtain local and global contextual feature information of wheat seedlings. It introduces a density-enhanced encoder module to improve the feature extraction ability of the model. The total loss function is constructed by combining counting loss, classification loss, and regression loss, reducing the technical difficulties of false positives and false negatives in wheat seedling detection. From the above experimental results, it can be seen that compared with other mainstream counting models, the DM_IOC_fpn model proposed in this study achieves a better balance between counting accuracy and efficiency of wheat seedlings, providing a feasible solution for real-time wheat seedling detection in the field.

The performance evaluation of this study is mainly based on the GPU environment, which to some extent limits its direct reflection of the applicability of the model on low-power edge devices such as drones. Due to the limitations of edge processors in terms of computing power, memory, and power consumption, the real-time inference capability of the model on such platforms still needs further verification. In future work, we will focus on exploring model lightweighting and compression strategy for the target edge configuration file Jetson Xavier, in order to reduce the computational complexity and storage requirement of the model. At the same time, the air-ground collaboration architecture can be considered, which uses edge device for real-time feature extraction and preliminary computation, while transferring complex inference task to the ground or cloud for processing, thereby ensuring real-time performance while reducing energy consumption. Through the exploration of the above direction, the adaptability and application value of the model on edge device will be further improved.

In this study, the wheat seedling image was obtained based on the UAV platform, and the accurate detection and counting of wheat seedling was realized based on the counting method of deep learning regression density map. From the perspective of result efficiency, the method proposed in this paper can replace the traditional manual counting, which not only saves time and labor cost, but also provides reliable counting results, and provides important data support for wheat intelligent breeding and planting. However, there are still many problems in the research of plant counting on the wheat seedling image taken by UAV. The wheat seedling detection model proposed in this study demonstrate good detection performance during the seedling stage, it still has certain limitations. These mainly include: (1) The model proposed in this study is specifically designed for the wheat seedling stage and has limited adaptability to other crops. It’s cross-crop generalization ability needs further optimization to meet the needs of cross-crop target detection tasks. (2) As wheat progresses from the seedling stage to the tillering stage and eventually heads and bears fruit, the plant structure and canopy morphology undergo significant changes. The follow-up work of this model will focus on improving its generalization ability, so that it can be migrated to target detection of different crops or growth stage. However, this model is mainly suitable for the seedling stage and lacks adaptability to subsequent growth stages and complex field scenarios. Nevertheless, the method proposed in this study still demonstrates some transferability in the generalization test of the WEDD dataset, indicating that its feature extraction and dense target recognition modules have good adaptability and robustness. (3) In large-scale breeding experiments, the model proposed in this study still faces issues such as insufficient algorithm generalization ability and poor transferability, which to some extent limit its potential value in high-throughput phenotypic data acquisition and application.

## Conclusion

In order to solve the problems of low precision and poor generalization of wheat seedling number detection model in the field, wheat seedling number detection model DM_IOC_fpn combining local and global features was proposed, which realized the accurate detection and counting of wheat seedlings. By building a double branch structure in the IOCFormer model, the local and global context characteristics of wheat seedlings are obtained; The density enhancement encoder module is introduced to improve the network structure and enhance the ability of network feature extraction; The total loss function is constructed by counting loss, classification loss and regression loss, which reduces the technical problems of false and missed detection caused by small wheat seedlings.

1. The experiments show that DM_IOC_fpn can accurately detect small target wheat seedlings, and the MAE and RMSE of self-built wheat seedling image dataset are 2.91 and 2.23, respectively, which are 1.78 and 1.04 lower than the original IOCFormer. Compared with other advanced counting models, DM_IOC_fpn realizes high-precision wheat seedling detection and counting, and can meet the requirements of wheat seedling detection in complex field environment.
2. The automatic counting results of wheat seedling in the actual field environment showed that DM_IOC_fpn can accurately predict number of wheat seedling, break the efficiency bottleneck faced by the traditional artificial number of seedlings, improve the accuracy and efficiency of wheat seedling data acquisition, better solve the problems of mutual occlusion and overlapping of small target wheat seedling, and improve the applicability in complex field environment.

Although the method proposed in this study has better counting performance compared to other methods, there are still some issues that need to be addressed. Future research work needs to further improve and refine relevant theories and methods, the difficulties and challenges are as follows: (1) Based on the new data feedback collected in real time, the dynamic update mechanism of the model is constructed to further optimize the wheat seedling network architecture, and comprehensively improve the generalization ability and accuracy of the wheat seedling network in complex scenes; (2) In view of the problems of the existing target detection model, such as large number of parameters, high consumption of computing resources, and difficult deployment in mobile devices, we urgently need to develop a lightweight wheat seedling detection model, so as to achieve real-time and accurate detection of wheat seedling number.

## Data Availability

The raw data supporting the conclusions of this article will be made available by the authors, without undue reservation.
